# Cytokines in the Brain and Neuroinflammation: We Didn’t Starve the Fire!

**DOI:** 10.3390/ph15020140

**Published:** 2022-01-25

**Authors:** Jan Pieter Konsman

**Affiliations:** UMR CNRS 5287 Aquitaine Institute for Integrative and Cognitive Neuroscience, University of Bordeaux, 146 Rue Léo Saignat, 33076 Bordeaux, France; jan-pieter.konsman@u-bordeaux.fr

**Keywords:** brain, cytokines, experimental allergic encephalomyelitis, infection, mental disorders, neuroinflammation, sepsis-associated encephalopathy, stress

## Abstract

In spite of the brain-protecting tissues of the skull, meninges, and blood-brain barrier, some forms of injury to or infection of the CNS can give rise to cerebral cytokine production and action and result in drastic changes in brain function and behavior. Interestingly, peripheral infection-induced systemic inflammation can also be accompanied by increased cerebral cytokine production. Furthermore, it has been recently proposed that some forms of psychological stress may have similar CNS effects. Different conditions of cerebral cytokine production and action will be reviewed here against the background of neuroinflammation. Within this context, it is important to both deepen our understanding along already taken paths as well as to explore new ways in which neural functioning can be modified by cytokines. This, in turn, should enable us to put forward different modes of cerebral cytokine production and action in relation to distinct forms of neuroinflammation.

## 1. Introduction

The brain is one of the bodily organs that is peculiar when it comes to immune responses to injury and infection. First of all, in many animal species, the brain is relatively protected from penetrating injury by the skull bone and the fibrous membranes of meninges surrounding it [1]. Although the concentration of nervous tissue in the head region of animals (cephalization) is evolutionarily ancient, the appearance of an ossified cavity containing the brain is more recent [2]. Indeed, for much of evolutionary history, the brain was protected by an exoskeleton. However, ever since the appearance of a skull bone, a certain correspondence in changes affecting the brain and those of the skull can be observed in the evolution and development of species [3,4]. This can be explained by positing that the skull also enables new feeding and sensory modalities besides providing protection of the brain [4].

Second, the so-called blood-brain barrier (BBB), formed by tight junction molecular bridges between, little fluid and particle uptake by, and low cellular passage of brain endothelial cells limits the entry of many infectious microorganisms into the central nervous system of many vertebrates [5,6]. Interestingly, BBB properties can also be found in some insects, including Drosophilia, but are then related to the structural organization of glial cells [7,8]. Furthermore, these glial cells express junction molecules and chemoprotective xenobiotic-excluding transporters, in addition to having the capacity to promote immune cell infiltration [9,10,11,12,13]. BBB properties therefore seem conserved between invertebrates and vertebrates by the expression of similar molecules, albeit in different brain cells, and may have been favored during evolution because they allowed for a more stable ionic and chemical extracellular brain environment [7].

### 1.1. Brain Cytokine Production and Action in Neurological Conditions: Immune Privilege Transformed

Nevertheless, and in spite of these brain-protecting tissues, some forms of head injury or infectious microorganisms can result in drastic changes in brain function and behavior as some historical examples of construction site accidents [14] and boxing- and football-related injuries [15,16], as well as the sequelae of some infectious diseases [17,18,19,20,21] illustrate. Notably, some of the infectious microorganisms that induce neurological symptoms in their hosts are capable of crossing the BBB by interacting with some of the molecules these interfaces express or by infecting immune cells that cross them [5,22,23,24]. Furthermore, brain cells, and in particular glial cells, have long been known to be capable of producing the pro-inflammatory cytokine interleukin-1 (IL-1) in response to administration of bacterial lipopolysaccharide (LPS) or local injury [25,26,27]. Interestingly, while introduction of bacterial fragments in the skin gives rise to full-blown inflammatory responses, including immune cell infiltration and the mutually-reinforcing production of the pro-inflammatory cytokines IL-1beta and tumor necrosis factor (TNF)-alpha, this is less the case in the brain parenchyma [28,29]. Thus, even though the skull, meninges, and BBB provide protection, injury to or infection of the brain tissue does occur and can lead to some inflammatory responses.

These latter observations need to be interpreted in the historical context of the idea that the brain has a privileged status regarding immunity. This idea was put forward to account for the relative lack of immune responses to tissue transplants or tumors in the CNS [30,31,32,33,34]. However, findings obtained in multiple sclerosis patients and in its animal model experimental allergic encephalomyelitis (EAE) clearly indicated that it was possible to observe immune and inflammatory responses in the brain parenchyma [35]. Moreover, from the mid-1980s onwards, the characteristics of the immune-privileged status and the conditions under which it applied became better known [36,37,38,39,40]. Thus, the observations that local injection of bacterial fragments or pro-inflammatory cytokines into the brain parenchyma gives rise to less neutrophil infiltration and cytokine induction as compared to other tissues became part of the further characterization of the immune privileged status of the CNS. It is clear, however, that its immune privileged status should not be understood as the brain being incapable of mounting immune responses [41].

While the role of brain pro-inflammatory cytokines in animal models of neurological conditions has often been guided by the hypothesis that these contribute to neuropathological processes, research findings clearly indicate that this needs to be nuanced. In the following discussion, the focus will be on IL-1, but with the idea that similar findings exist regarding TNF. Early clinical studies have shown that CSF IL-1 beta concentrations correlate with cerebral pathology in onset multiple sclerosis [42,43]. These findings have been corroborated by the detection of increased cerebral expression of IL-1beta as well as of the IL-1 receptor antagonist at sites of starting demyelination during EAE [44]. Furthermore, brain overexpression of the IL-1 receptor antagonist or IL-1 receptor deficiency reduces cerebral chemokine production, macrophage infiltration, and EAE disease severity [45,46]. More specifically, the brain endothelial selective knock-down of signaling IL-1 receptors lowers adhesion molecule expression and cerebral immune cell infiltration and clinical scores in EAE [47,48].

Already in the early 1990s, CSF IL-1beta levels were reported to be higher in Alzheimer Disease (AD) than in Multiple Sclerosis patients [49]. This, along with the increased expression of IL-1 and IL-1 converting enzyme in the temporal brain lobe of patients with AD [50,51], gave rise to the idea that pro-inflammatory cytokines play a role in AD. Interestingly, genetically-modified mice expressing the amyloid precursor protein with mutations found in familial forms of AD also show increased IL-1beta expression in glial cells in close proximity to amyloid plaques [52,53]. While the main assumption was that IL-1beta would play a detrimental role in AD, it was actually found that increased brain IL-1beta production by hippocampal IL-1beta overexpression in AD mouse models reduces amyloid plaque load, even though it increases tau pathology [54,55,56]. However, hippocampal transplantation of IL-1 receptor antagonist-expressing neural precursor cells or intracerebroventricualar administration of the IL-1 receptor antagonist has been shown to mitigate cognitive deficits in a mouse AD model and to reduce hippocampal plaque load [57]. While these findings indicate that increased brain pro-inflammatory cytokine production should not be simply assumed to play a detrimental role in the symptoms and disease processes of neurological condition, they also suggest that presumed disease processes and symptoms are not always tightly linked.

### 1.2. Brain Cytokine Production and Action in Physiology, Behavior, and Cognition

Interestingly, from the mid-1980s onwards, it has repeatedly been shown that the brain not only responds to local injury or infection with increased production of IL-1, but also during systemic inflammation induced by the intravenous or intraperitoneal administration of bacterial LPS [58,59,60,61,62]. Although systemic administration of bacterial LPS is often considered to be an animal model of bacterial sepsis, it is important to keep in mind that it does not mimic the hemodynamic phases observed in clinical sepsis. Importantly, cecal ligature and puncture (CLP) in rodents does reproduce clinical sepsis-associated hemodynamic phases and results in increased cerebrospinal fluid concentrations and brain expression of IL-1beta and TNF-alpha [63,64]. Finally, sepsis giving rise to fatal childhood malaria and in premature babies is also accompanied by elevated IL-1beta and TNF-alpha levels in the brain parenchyma and cerebrospinal fluid levels [65,66]. Therefore, brain pro-inflammatory cytokine production clearly not only occurs in response to local insults of central nervous tissue, but also as a result of systemic inflammation.

Although it was initially hypothesized that brain IL-1 plays a role in fever induction, it was first shown to contribute to the increased time that rodents injected with bacterial fragments spent sleeping [67,68]. Subsequently, central IL-1 action has also been reported to play a role in bacterial LPS- and CLP-induced systemic inflammation-associated fever and sickness behavior (reduced food intake and social interactions) [69,70,71,72]. Because fever and low food intake, as well as reduced activity and increased sleep, can be considered adaptive when the organism is facing a bacterial infection [73], these findings can be interpreted to suggest that IL-1 action in the brain activates specific circuits that lead to an integrated physiological and behavioral response to overcome infection. One of the outstanding questions in this respect is where in the brain IL-1 acts to bring about these physiological and behavioral changes.

Learning and memory can also be affected by the peripheral injection of bacterial LPS, but this often seems to depend on the test and experimental conditions used [74]. Although central injection of IL-1 at some doses can impair learning and memory processes [74,75], it is not established that the brain action of endogenous IL-1 mediates the effects of peripheral bacterial LPS injection on learning and memory. In fact, it has been shown that central administration of the IL-1 receptor antagonist, at a dose that attenuates peripheral bacterial LPS-induced fever, does not affect associative learning between a taste and LPS injection [76]. However, intracerebroventricular administration of IL-1 receptor antagonist has been reported to mitigate the detrimental effect of sepsis on aversive memory in the step-down inhibitory avoidance test [77]. Thus, the role of brain IL-1 in mediating bacterial infection-associated cognitive alterations remains to be further clarified.

Importantly, increased brain IL-1 production does not only occur in response to the detection of bacterial fragments. Indeed, cerebrospinal fluid concentrations of IL-1 also increase during sleep in comparison to the wake state of animals, even in the absence of exposure to microbial fragments [78,79]. Moreover, the central inhibition of IL-1 action has been shown to reduce sleep and its rebound after sleep deprivation [68,80,81]. Altogether, it thus seems that IL-1, a mediator classically associated with the immune system, plays a role in the physiological regulation of sleep in the brain.

Another potential physiological role for brain IL-1 concerns learning and memory. The first indication of this was provided by a paper showing that long-term potentiation (LTP), a mechanism thought to underlie some forms of learning, increases the expression of IL-1beta in the hippocampus and that administration of the IL-1 receptor antagonist impairs LTP maintenance [82]. Interestingly, LTP also increases hippocampal IL-6 expression, and antagonizing its action results in prolonged LTP and improved memory in a spatial alternation paradigm [83]. Beyond LTP, fear-motivated context-dependent inhibitory avoidance training has been shown to increase the expression of IL-1 alpha in the hippocampus, and local overexpression of the interleukin-1receptor antagonist has been found to improve the retention scores of this task [84]. Contextual learning of fear-conditioned freezing also increases hippocampal IL-1 beta expression with the central production of IL-1 receptor antagonist reducing freezing in this test [85]. In addition, this latter study also showed that central administration of a low dose of IL-1beta facilitates context-conditioned freezing, whereas a higher dose impairs this response [85]. Thus, there is good evidence that central IL-1 beta plays a dose-dependent role in contextual fear conditioning and hippocampal synaptic function [74,86]. Finally, other work has indicated that Morris water maze training increases the expression of the so-called anti-inflammatory cytokines IL-4 and IL-13 expression in the brain meninges and that genetic deficiency of either of these cytokines affects cognitive performance in this maze [87,88]. In summary, as for the role of brain IL-1 in systemic inflammation-associated changes in cognition, its physiological role in mediating cognition still needs to be further clarified.

### 1.3. ‘Psychological Stress’—Associated Cytokine Production

Although the general term ‘stress’ may be considered a vague ‘umbrella concept’, some distinctions of stress categories may prove useful. Indeed, based on the eliciting stimuli or conditions, one can distinguish between systemic, homeostatic, or physiological stressors and neurogenic, emotional, or psychological stressors. Infection-induced systemic inflammation can thus be considered a homeostatic-physiological stressor that is accompanied by pro-inflammatory cytokine production and action in the brain, at least in the experimental model consisting of peripheral LPS administration (see above).

Interestingly, a relationship between chronic emotional-psychological stress and pro-inflammatory cytokine production has also been proposed. One of the first findings indicating such a link was that of chronic caregiver stress being associated with increased expression of transcripts with response elements for NF-kappaB, a pro-inflammatory transcription factor, in circulating monocytes [89]. Many studies have subsequently looked at circulating markers and have reported increases in the plasma IL-6 of the elderly taking care of a spouse with a chronic medical condition, persons with a low socioeconomic status, victims of childhood abuse or maltreatment, and of patients with depression in comparison to respective control groups [90]. In addition, studies on animals indicated that chronic unpredictable mild stress increases serum IL-6, IL-1beta, and TNF-alpha [91], while repeated social defeat stress results in higher circulating IL-6 concentrations [92,93]. Interestingly, chronic social defeat stress has also been shown to result in increased IL-1beta synthesis in the brain [94]. While it seems clear that some stressful situations can give rise to increased cytokine production, including in the brain, the extent to which some of these stressors can be considered as purely psychological is, at present, unclear.

## 2. Context-Dependent Neuroinflammation and Brain Cytokine Production

Over the past four decades, it has clearly been shown that inflammatory and immune responses can occur in the brain, but also that these may be different from peripheral tissues and thus confer an immune-privileged status to the brain. As mentioned above, in response to injection of the same amount of bacterial lipopolysaccharide (LPS) fragments, the skin displays full-blown local inflammatory responses, whereas the parenchyma of the central nervous system shows delayed cellular infiltration with minimal recruitment of neutrophils [28]. However, the brain meninges, circumventricular organs, and choroid plexus display LPS-induced responses that resemble those observed in the skin [28]. Yet, after severe CNS injuries, such as stroke, the brain parenchyma can also show inflammatory responses that are reminiscent of the hallmarks of peripheral inflammation. Such responses have historically been named neuroinflammation [95]. Thus, it has been proposed that neuroinflammation refers to four signs, namely increased cytokine production, activation of microglia, peripheral immune cell recruitment, and local tissue damage [95]. However, over the years, the phenomena for which this term has been used go well beyond central nervous tissue responses to severe injuries. Indeed, the term neuroinflammation has regularly been employed after the detection of one single hallmark in a wide variety of physiological and psychological stressors [95,96,97].

As pointed out above, systemic inflammation induced by peripheral administration of bacterial LPS, as well as in animal models of sepsis, are accompanied by pro-inflammatory cytokines production, in particular at the blood-brain interfaces of the meninges, circumventricular organs, and choroid plexus. Moreover, both the peripheral administration of bacterial LPS and experimental sepsis models result in morphological signs of microglial activation [98,99,100]. Furthermore, both procedures induce immune cell infiltration of the brain [101,102,103,104,105,106]. Finally, regarding cerebral tissue damage, both BBB breakdown and neuronal death have been studied. While leakage of circulating molecules into the brain parenchyma after systemic injection of bacterial LPS or induction of sepsis has been repeatedly reported [107,108], it is important to bear in mind that BBB breakdown does not seem to be necessary for signs of sickness or encephalopathy [109,110,111,112]. While clinical septic shock, experimental sepsis, and the systemic administration of high doses of bacterial LPS can all lead to signs of neuronal apoptosis [113,114,115], this is not necessarily the case for moderate doses of LPS administered in adult animals [116]. Taken together, these findings clearly indicate that systemic inflammation during bacterial sepsis or induced by high doses of bacterial LPS can result in *bona fide* neuroinflammation.

Regarding the effects of psychological stress, it has already been mentioned above that chronic social defeat increases brain IL-1beta synthesis. As for glial activation, there is evidence that fear conditioning, chronic foot shock, restraint, and social defeat in adult rodents lead to microglial activity in the brain based on immunohistochemcial staining of the microglial specific marker ionized calcium binding adaptor molecule 1 (Iba-1) [117,118]. In addition to microglial activation, chronic social defeat also enhances recruitment of mononuclear immune cells to the brain perivascular spaces [119,120] and results in local breakdown of the BBB [121]. Altogether, the available evidence suggests that features of neuroinflammation, including increased pro-inflammatory CNS cytokine expression, glial activation, brain recruitment of immune cells, and breakdown of the BBB, are associated with some stress conditions in rodents, in particular with chronic social defeat. It remains to be seen, however, if neuroinflammation occurs more generally in stressful conditions beyond that induced by chronic social defeat.

In spite of the conclusion that neuroinflammation occurs during severe sepsis-like systemic inflammation and some experimental stressful conditions, it is important to keep in mind that it is based on the consideration of a whole corpus of published articles and that there are important differences in the type of neuroinflammation between these conditions. This is especially important in a context where some individual articles make conclusions regarding the occurrence of neuroinflammation when addressing only one feature, for example microglial activation, in a particular condition [97,117].

## 3. Novel Insights into Brain Cytokine Production and Action

In what follows, different aspects of cerebral cytokine production and action, as recently reported in Pharmaceuticals, will be discussed. If, in the previous sections, the focus was mainly focused on IL-1 to more clearly set the different contextual scenes, these articles also assess many other cytokines. The discussion will start within the context of neurology and progressively move to physiology and behavior before touching on more psychiatric aspects.

As alluded to above, MS and its animal model EAE have been studied extensively with regard to the role of cytokines and neuroinflammation. Although intercellular communication via cytokine release into the extracellular cerebral environment has been mostly addressed, there are other ways in which different brain cell types can interact. For example, gap junctions, made up of connexin molecules, connect different glial cell types and enable the exchange of small molecules. Interestingly, the expression of connexins is increased in MS [122], and mutations in connexin molecules are associated with myelin-related disorders, such as X-linked Charcot–Marie–Tooth disease [123]. While this suggests that connexins could play a role in MS pathophysiology, mice that are genetically-deficient in individual connexins do not spontaneously show signs of demyelination [124,125]. However, and as shown previously by the group of Kleopa, these mice are more susceptible to EAE with higher clinical scores and more severe neuroinflammation and demyelination [126]. In a follow-up study, published in Pharmaceuticals, this group studied CNS blood-spinal cord interfaces; immune cell infiltration; and the expression of certain adhesion molecules, chemokines, and cytokines at different time points prior to and after EAE onset in connexin-deficient mice. It was thus confirmed that mice deficient in connexin-47, expressed mainly by oligodendrocytes, have a faster-appearing and more severe disease with higher immune cell infiltration, along with lower blood spinal cord barrier-associated tight junction molecule expression already before disease onset [127]. Interestingly, none of these early phenomena were found to be accompanied by a significant increase in the CNS expression of the adhesion molecules, chemokines, and cytokines considered, even though changes were observed later during the disease cause [127]. These findings strongly encourage researchers to address factors other than the usual suspects in the context of emerging neuroinflammation.

In the preceding sections, it was pointed out how cerebral cytokine production and neuroinflammation not only occur in response to local insult of the CNS, as in MS or EAE, but also during systemic inflammation secondary to peripheral infection. However, almost all the findings indicating brain cytokine production and other signs of neuroinflammation during system inflammation have been obtained in animal models of bacterial infection. It is thus important to establish to what extent these phenomena also occur during viral infections. As indicated by Bohmwald et al. in Pharmaceuticals, “increased levels of pro-inflammatory molecules, such as IL-1beta, IL-6, and TNF-alpha, have been detected in the CSF of patients showing neurological alterations due to influenza virus infection” [128] (p. 4). Moreover, these clinical findings are corroborated by experimental studies showing increased cerebral expression of pro-inflammatory cytokines inoculated peripherally with the influenza virus [128]. Therefore, while it seems clear that pro-inflammatory cytokines are being produced in the brain in response to a peripheral influenza infection, the sites of the action and the role(s) of these cytokines in mediating viral infection-associated symptoms remain to be clarified.

In an effort to deepen our understanding of the neuroinflammatory mechanisms relevant to sepsis-associated encephalopathy, Moraes et al. offer a review, published in Pharmaceuticals, focusing on the activation of microglial cells by danger-associated molecular patterns and pathogen-associated molecular patterns and their consequences in the context of brain dysfunction during sepsis. These authors relate findings of several postmortem studies showing increased cerebral expression of CD68, which can be taken as an indicator of microglial activation, in patients who had succumbed to sepsis [129,130,131]. Furthermore, Moraes et al. hypothesize that “IL-1beta derived from activated microglia is responsible for the synaptic deficits observed in sepsis” [132] (p. 12).

Obviously, bacterial sepsis can induce the production of many cytokines, including in the brain. In an animal model that attempts to mimic the massive release of bacterial LPS sometimes observed in clinical sepsis [133], Peek et al., in their article published in Pharmaceuticals, focused on the High Mobility Group Box-1 Protein (HMGB-1), a nuclear DNA-binding protein that alters the structure of chromatin, but which can serve as a danger-associated molecular pattern or alarmin and mimic pro-inflammatory cytokine activity when present in the extracellular space [134]. These authors relate studies showing that circulating concentrations of HMGB-1 are increased during severe clinical sepsis and septic shock [135] and several hours after the peripheral administration of high doses of LPS to rodents [136]. Interestingly, oxidative stress favors the formation of disulfide HMGB-1, which can, just like bacterial LPS, activate the toll-like receptor 4 (TLR4) [134]. Given that TLR4 is preferentially expressed in brain circumventricular organs lacking a functional BBB [137], Peek et al. set out to study the effects of disulfide-HMGB-1 on the neuro-glial cell cultures of the area postrema, the brainstem circumventricular organ, as well as the effects of prior bacterial LPS-induced inflammation on the response of area postrema cells to HMGB-1. While they confirmed that peripheral administration of LPS leads to a sustained increase in circulating HMGB-1, they also provided *in vivo* evidence of LPS-induced nucleus-to-cytoplasm translocation of HMGBI in the hypothalamus and area postrema, which can be interpreted to represent a step in HMGB-1 release into the extracellular environment [138]. This latter effect could be reproduced *in vitro* by exposing area postrema cultures to LPS. Mimicking HMGB-1 release by exposing area postrema cultures to disulfide HMGB-1 resulted in increased nuclear factor-kappaB staining in cell nuclei, indicating the activation of a pro-inflammatory intracellular signaling cascade and IL-6 release [138]. Finally, Peek et al. obtained evidence that prior LPS exposure primes area postrema cultures’ responsiveness to subsequent HGMB1 [138]. Thus, HGMB1 can be hypothesized to play a role in the sustained effects of bacterial LPS on the brain.

The contribution of Kvivik et al. in Pharmaceuticals on the detection of HMGB-1 in biological fluids is relevant for that of many classic cytokines as proteins with molecular weights between 6 and 70 kDa [139]. These authors hypothesize that HMGB-1 could play an important role in mediating sickness behavior, but they were confronted with the difficulties of current available methods, such as autoantibodies or plasma proteins interfering with the detection of HMGB-1 by enzyme-linked immunosorbent assay. They, therefore, set out to develop an antibody-free liquid chromatography coupled with a tandem mass spectrometry-based detection method for HMGB-1. In particular, Kvivik et al. showed that, notwithstanding suboptimal recovery, the method developed enabled “the identification of several unique HMGB-1 peptides” [140] (p. 12). This accomplishment, in turn, has the potential to allow for “the measurement of different redox variants” in the future [140] (p. 12).

In the context of the study of sickness behavior, Chaskiel et al. tested the role of IL-1 receptor-expressing cerebral perivascular macrophages in mediating reduced food intake and carried out exploration after central systemic administration of IL-1beta. The authors showed previously that the peripheral IL-1beta-induced reduction of food intake is, in part, mediated by IL-1 receptors in the arcuate hypothalamus [141] and wondered if another part could be played by brain perivascular macrophages, also known to express IL-1 receptors [142]. To test the role of IL-1 receptor brain macrophages, the work of Chaskiel et al., published in Pharmaceuticals, employed IL-1 coupled to the ribosome toxin, saporin, and administered this conjugate into forebrain ventricles, from where molecules spread preferentially through perivascular spaces. This intervention effectively reduced the number of CD163-positive perivascular macrophages in a forebrain circumventricular organ as well as along vessels forming a functional BBB, but it did not affect the reduction of food intake after subsequent IL-1beta administration [143]. Therefore, while these findings clearly indicate that brain perivascular macrophages do not mediate IL-1-induced sickness behavior in rats, it remains possible that these cells play an important role in bringing about sickness behavior as cerebral sources of IL-1 beta production in response to circulating bacterial fragments.

Finally, two articles in *Pharmaceuticals* address the cerebral actions of cytokines in the context of mental disorders. The first, by Ivanovska et al., is a review focused on the chemoattractant cytokine eosinophil chemotactic protein CCL-11, also called eotaxin, which binds to CCR3 [144]. Of particular interest to the topic of cerebral cytokine production action is the finding that helminth infection can result in increased CSF CCL-11/eotaxin concentrations [145,146]. Other interesting features of this cytokine are that blood CCL-11/eotaxin concentrations increase with age [147] and that circulating CCL-11/eotaxin is likely to be transported into the CNS and accumulate there [148]. After a review of the relevant literature, Ivanovska et al. conclude that: “Plasma levels of CCL-11 are increased not only in schizophrenia and age-related cognitive impairments, but also in some patients with mood disorders” [149] (p. 10). Although the conditions in which circulating CCL-11/eotaxin is reported to be increased seem, at first sight, to be very different, they may have much in common “accelerated aging” [150] (p. 1). Scheiber et al. address similar questions by measuring plasma and CSF cytokines and the surface antigen expression of circulating immune cells. Their results, to be reported in *Pharmaceuticals*, show that CSF IL-8 concentrations were higher than in the blood of patients with affective spectrum disorder or schizophrenic spectrum disorder. In contrast, plasma IL-1 beta concentrations were found to be higher than those in CSF for patients with schizophrenic spectrum disorder (Scheiber et al., *Pharmaceuticals*, under review). In light of these findings suggesting increased IL-8 brain production in affective spectrum and schizophrenic spectrum disorders and given the neutrophil chemoattractant role of this cytokine [151], one would like to see future studies addressing CSF neutrophil counts in these mental disorders. If this were to be true, then the case for some form of neuroinflammation accompanying certain mental disorders would be considerably strengthened.

## 4. Conclusions

The aim of this review, as part of the Special Issue of *Pharmaceuticals* on “cerebral production and action of cytokines”, was to better understand cytokine production and action in the brain beyond their involvement in local immune responses to an insult or infection of the CNS. One domain in which research into brain cytokine production and action has been particularly active over the past decades is that searching to explain the occurrence of non-specific disease symptoms involving CNS-regulated physiology and behavior, such as fever, increased sleep, and reduced food intake, after the systemic detection of bacterial fragments. An important outstanding question in this respect is how to distinguish sickness physiology and behavior and the underlying neuroimmune communication pathways from the neuroinflammatory processes involved in sepsis-associated encephalopathy. Another domain of research addressing the role of brain cytokine production and action concerns the regulation of some important functions, including sleep and cognition. While the majority of the work on brain cytokine production and action has historically concerned a couple of pro-inflammatory cytokines, such as IL-1, it is important to realize that cytokines comprise many different biologically active molecules and include interferons, interleukins, and chemokines, but also, for example, adipokines. Thus, several new perspectives have arisen regarding cytokine brain production and action that surpass classic neuroimmunology and neuroinflammation and seem closer in spirit to integrative physiology and behavior. These new perspectives can, in the long run, be expected to give rise to new therapeutic avenues.

## Data Availability

Not applicable.
